# EEOA: Cost and Energy Efficient Task Scheduling in a Cloud-Fog Framework

**DOI:** 10.3390/s23052445

**Published:** 2023-02-22

**Authors:** M. Santhosh Kumar, Ganesh Reddy Karri

**Affiliations:** School of Computer Science and Engineering, VIT-AP University, Amaravathi 522237, Andhra Pradesh, India

**Keywords:** electric fish optimization, earthworm optimization algorithm, internet of things, HPC2N, CEA-CURIE

## Abstract

Cloud-fog computing is a wide range of service environments created to provide quick, flexible services to customers, and the phenomenal growth of the Internet of Things (IoT) has produced an immense amount of data on a daily basis. To complete tasks and meet service-level agreement (SLA) commitments, the provider assigns appropriate resources and employs scheduling techniques to efficiently manage the execution of received IoT tasks in fog or cloud systems. The effectiveness of cloud services is directly impacted by some other important criteria, such as energy usage and cost, which are not taken into account by many of the existing methodologies. To resolve the aforementioned problems, an effective scheduling algorithm is required to schedule the heterogeneous workload and enhance the quality of service (QoS). Therefore, a nature-inspired multi-objective task scheduling algorithm called the electric earthworm optimization algorithm (EEOA) is proposed in this paper for IoT requests in a cloud-fog framework. This method was created using the combination of the earthworm optimization algorithm (EOA) and the electric fish optimization algorithm (EFO) to improve EFO’s potential to be exploited while looking for the best solution to the problem at hand. Concerning execution time, cost, makespan, and energy consumption, the suggested scheduling technique’s performance was assessed using significant instances of real-world workloads such as CEA-CURIE and HPC2N. Based on simulation results, our proposed approach improves efficiency by 89%, energy consumption by 94%, and total cost by 87% over existing algorithms for the scenarios considered using different benchmarks. Detailed simulations demonstrate that the suggested approach provides a superior scheduling scheme with better results than the existing scheduling techniques.

## 1. Introduction

The Internet of Things (IoT) is a modern invention that has a significant impact on information and communication technologies (ICT). The IoT and its related technologies, such as machine-to-machine (M2M) advancements, expand Internet access to a variety of gadgets and household objects (such as artifacts, devices, automobiles, and residential complexes), enabling them to carry out a range of applications and functions (e.g., vehicular networking, energy management, traffic control, medical treatment, and health care) [1,2,3,4]. Incredible amounts of data are being produced by these smart devices, which must be maintained, processed, and analyzed to gain important ideas and make them accessible to end users and/or client software [5,6].

Fog computing has developed as a unique framework to complement cloud computing and the fast evolution of the IoT [7,8,9]. To address the issue of data latency, the fog attempts to extend the cloud to the network’s edge, near where IoT data are generated. For delay-sensitive, energy-efficient, high-privacy, and security applications, data are processed at edge nodes, and the high volumes of data are transferred to the cloud for processing and storage. Both computing paradigms are very important for all types of IoT data [10]. In addition to that, the edge-to-cloud continuum improves the quality of service (QoS) [11].

Resources in the fog can be globalized, just like in cloud computing. In light of this, a fog node might be a virtual machine (VM) [12,13,14]. The processing capacity of the fog resources is often constrained, in contrast to the cloud computing model.

The QoS and financial cost are both greatly impacted by the work scheduling issue in collaborative fog-cloud computing systems [15,16,17]. There are numerous latency-sensitive and latency-tolerant IoT applications with various requirements in the real world. This makes organizing and scheduling them more difficult. As a result, we must offer an effective method of task scheduling in this situation [18,19,20]. Additionally, the cost of computation and communication for scheduling workflows is significant, especially for science-related workloads that deal with areas such as space exploration and the natural sciences, two of the most well-liked cloud platforms.

Service providers offer customers distinct functions in cloud computing at different prices. Usually, quicker resources are costlier than stronger [21,22]. The scheduler therefore has a range of resources for workflow, resulting in a range of runtimes and prices as well as a range of user limitations. The deadline makes sure that the procedure is finished in the allotted time. The cost constraint guarantees that the expense does not exceed the customer’s spending limit. The ideal answer balances these two demands [23,24,25]. Additionally, when defining the problem, the majority of the existing techniques fail to consider the system’s violation cost.

Therefore, in this study, we take into account the importance of tasks based on their cost, duration, and energy consumption when scheduling them in fog-cloud computing. To accomplish this, an efficient hybrid approach based on the electric fish and earthworm optimization algorithms, termed the electric earthworm optimization algorithm (EEOA), is created to perform cost and energy-conscious job scheduling in cloud/fog environments. The benefits of both metaheuristic algorithms are used to execute efficient scheduling while taking SLA requirements into account.

The following list summarizes the article’s contributions:

- To execute efficient task scheduling in cloud-fog environments, a hybrid evolutionary algorithm called EEOA is proposed. This algorithm combines the adaptive EOA technique with the fundamental EFO methodology to enhance convergence speed and searching explorations.
- Creating an energy-efficient cost model that takes into account the QoS requirements and energy requirements of IoT tasks sent for processing in a cloud-fog framework.
- Assessing the proposed scheduling method’s efficiency to other strategies on real-world workloads such as HPC2N and CEA-Curie in terms of makespan, task execution cost, and power consumption.

The remainder of the article is organized as follows. In Section 2, the study on existing work scheduling techniques is covered. The system design is detailed in Section 3 and Section 4 and describes the problem formulation and objective function. In Section 5, the suggested EEOA job scheduling algorithm is explained. The effectiveness of the EEOA technique is shown in Section 6. The conclusion and the work’s future focus are presented in Section 7.

## 2. Literature Review

To establish an effective scheduling method for integrating software applications in a cloud-fog framework, Arshed et al. [26] presented a genetic-algorithm-based scheduling technique. The suggested method first encodes the problem’s chromosome-based representation by taking into account the application and fog device attributes. The solutions were then evolved utilizing a variety of parameter modifications using genetic-algorithm-based crossover and mutation operators. In terms of run time, bandwidth, and financial cost, the suggested technique has been assessed and contrasted against existing techniques.

Ghafari and Mansouri [27] developed a task scheduling technique for the cloud system that maps workloads through the resources available using a grey wolf optimizer. This paper’s main objective was to reduce execution costs, energy use, and make-up time. This involves sending input tasks to the cloud system job queue. The VM controller subsequently received incoming tasks from the job queue. The VM management team then evaluates the resource situation. The tasks were assigned to the existing VMs if it was practicable to do so; otherwise, new VMs were generated.

Arshed and Ahmed [28] suggested the resource aware cost-efficient scheduler (RACE), which allocates the received application modules to fog systems that increase the utilization of resources at the fog layer and lower the cost in the cloud with the least amount of bandwidth utilization. There are two algorithms in this RACE. The arriving application modules were categorized by the module scheduler in RACE based on their computing and bandwidth needs and are subsequently positioned by a compare module.

Sindhu and Prakash [29] presented the CBTSA algorithm to symmetrically balance energy efficiency, cost, and task scheduling. First, this technique uses a directed acyclic graph to represent the priority of the workload that is accepted for processing. A node was chosen to process the workloads that had been given the highest priority. The tasks are handled on a fog or cloud node based on an optimal efficiency factor that yields the completion time, expense, and power. Along with the reinforcement learning algorithm, a Markov decision process was used to figure out where the best resources should go.

A multi-swarm particle swarm optimization (MS-PSO) technique was created by Subramoney and Nyirenda [30] to enhance the schedule of scientific activities in cloud-fog frameworks. The classic PSO has an early convergence issue that results in less-than-ideal outcomes. MS-PSO attempts to solve this issue. This method divided the particles into swarms, each with its own intellectual and socialization factors. It also developed a multi-criteria optimal task scheduling solution with four goals: load balancing for cloud and fog layers, price, power, and computation time. The GA-PSO, differential evolution, genetic algorithm, and conventional PSO methodologies were all used to assess the strategy’s effectiveness.

A multi-queue priority-based scheduling task was created by Fahad et al. [31] to perform a balanced task allocation for latency-sensitive fog applications and programs that can accept a certain degree of processing delay. This method classifies jobs as short or lengthy based on their burst time during execution. Each job category was maintained in its own task queue by the MQP algorithm, which also dynamically updates the preemption time slot value. It decreases reaction times for data-intensive applications in the fog computing environment, including both latency-sensitive jobs and those that are less latency-sensitive, thereby resolving the hunger issue for less latency-sensitive workloads.

Chhabra et al. [32] introduced a hybrid oppositional differential evolution-enabled WOA (h-DEWOA) technique to reduce task duration and energy consumption. It extends the capabilities of the traditional WOA method by incorporating the fitness-based balancing method, differential evolution, opposition-based learning, and chaotic maps. This leads to an adequate exploration/exploitation tradeoff, quicker convergence, and improved exploration throughout the algorithm’s execution. In addition, to enhance resource assignment, an allocation technique was incorporated into the h-DEWOA technique. The performance of scheduling methods was assessed using HPC2N and CEA-Curie workloads. To compare performance, two sequences of tests were performed. One uses WOA-based heuristics, and the other uses non-WOA-based metaheuristics.

Authors in [33] developed a task scheduling model which avoids local search problem and improves the scheduling performance in cloud environment by improving makespan, energy consumption. For this simulation, authors used improved cuckoo and OBL approaches by adding a differential evolution parameter to improve searching capability and thereby improvement of scheduling approach. Simulation carried out on Cloudsim and workload traces captured from HPC2N and evaluated against existing metaheuristic approaches and observed improvement over existing baseline approaches for above mentioned parameters. 

In [34], authors formulated a task scheduling mechanism to improve the Quality of Service and minimize energy consumption. For this to happen, authors used CSPSO approach by improving velocity of cuckoo search. This simulation carried out on Cloudsim by taking supercomputing work logs and compared over existing baseline approaches and from simulation results, it was revealed that FARA outperforms existing approaches by minimizing makespan, energy consumption and improves quality of service.

In [35], authors worked on scheduling process in cloud environment with HPC workloads and they addressed the problem of generating scheduling decisions in HPC cloud environment by addressing energy performance trade-off. They used genetic algorithm as their methodology to generate schedules for their parallel computational tasks. It was implemented on Cloudsim and they compared their model with existing baseline approaches and finally results revealed that their approach greatly minimizes energy consumption and improved the performance of the scheduler.

Electric fish optimization (EFO) is a meta-heuristic algorithm that functions based on the electrolocation and electrocommunication capabilities of electric fish [36]. Electric fish have the ability to use electrolocation to sense their surroundings and identify potential prey. Active electrolocation and passive electrolocation are the two main branches that explore the electrolocation capacity of electrified fish. This algorithm is mainly used to solve high-dimensionality problems, and task scheduling in fog computing is a highly dynamic scenario. Therefore, it can be helpful to solve task scheduling problems in fog computing. By using this electric fish optimization, the process in the solution space is balanced between local search and global search.

Wang, Gai-Ge et al. [37] proposed a bio-inspired metaheuristic algorithm for consistent and discrete constrained optimization problems. The earthworm’s beneficent effect on the natural world served as inspiration. Natural type 1 reproduction never produces hybrids between different species. It is possible to reproduce with multiple species using Reproduction 2. The EOA method can find optimal solutions on a global and a local scale.

From the above classification mentioned in the Table 1, we clearly observed that earlier authors used parameters such as makespan, energy consumption, and SLA-based trust parameters in cloud computing paradigms. In order to schedule tasks effectively in a fog environment, we proposed an effective cost and energy-concerned electric earthworm optimization algorithm (EEOA), which addresses makespan, total cost, and energy consumption by using EFO and EOA approaches. The proposed approach is really helpful in the real-time applications, i.e., smart cities, and it also helps greatly in scheduling latency sensitive applications such as vehicular networks.

## 3. System Architecture

**Figure 1:**
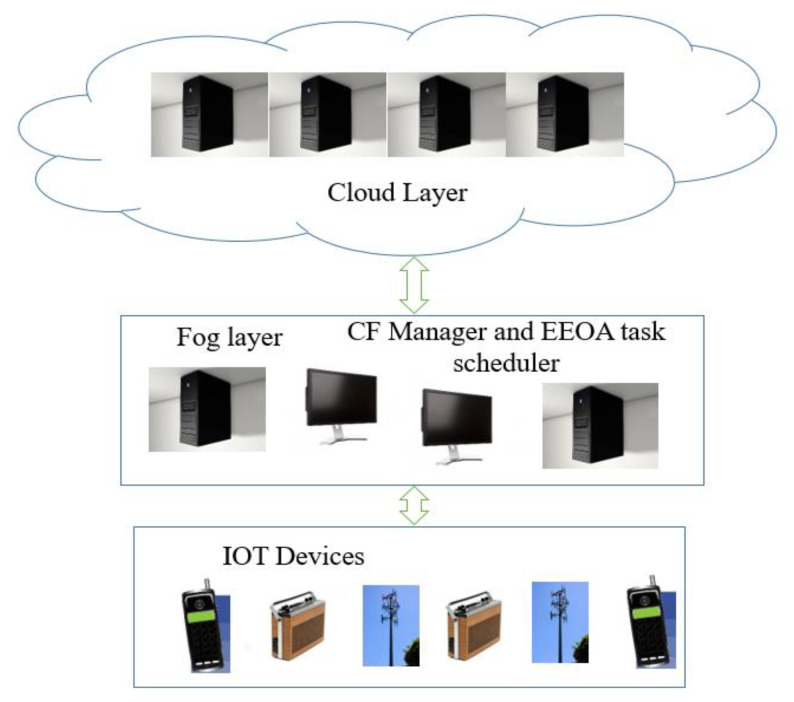
System Architecture.

Figure 1 depicts the three-layer architecture of the cloud-fog computing framework. The IoT devices (*i*_1_, *i*_2_, …, *i_n_*) that make up the first layer acquire data and deliver them to the immediate upper layer for processing. Fog nodes (*f*_1_, *f*_2_, …, *f_g_*) that are fitted with computers, mini-servers, and smart gateways make up the intermediate fog layer. With constrained computing, storage, and internet connectivity, each fog node serves as a smart device. The tasks that a fog node *Fg* receives are atomic and independent of one another; they do not contain any data that can be shared with tasks from other fog devices. Fog node F transmits data that cannot be handled locally to a distant cloud for additional processing and analysis. High-performance servers with adequate processing and storage capacity make up the top cloud layer, as depicted in Figure 1. Each server is made up of a number of virtual machines, such as (*VM*_1_, *VM_2_*, …, *VM_k_*). Each virtual machine has a memory and processing speed that define it.

Depending on the request made at the time of processing, data can transfer from fog to cloud as well as from cloud to fog. The fog layer has a task scheduler and a cloud fog manager (CFmanager), which collects all the resources and tasks. The job is transferred after the task-scheduling strategy decides whether it will be carried out in the cloud node or the fog. The task’s execution result is returned to the CF manager. All of the results are combined by the CF manager before being sent to the IOT systems. To create an effective job execution schedule, the proposed task-scheduling algorithm is installed in CFmanager.

## 4. Problem Formulation

**Figure 2:**
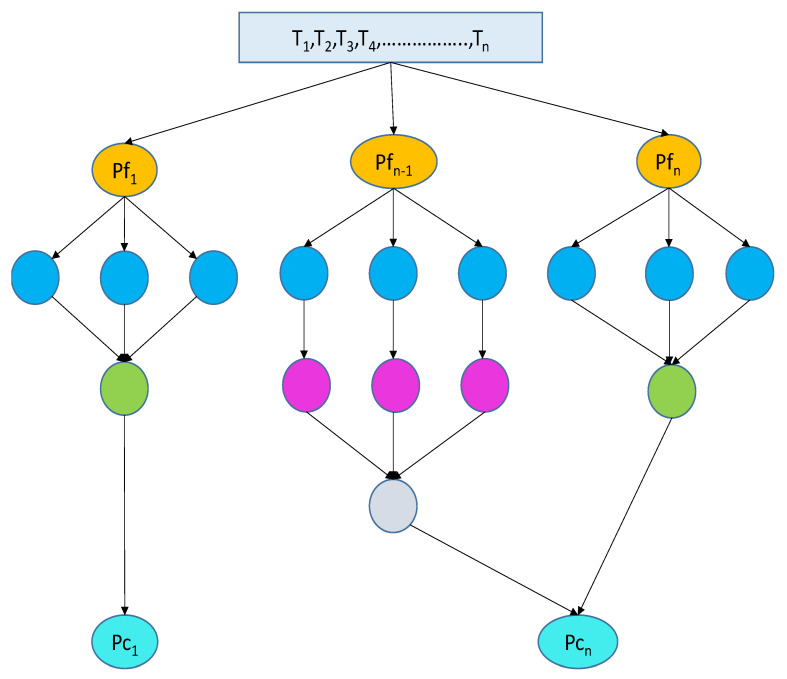
Cloud and fog workflow diagram.

The scheduling system is represented as a direct acyclic graph (DAG) as shown in Figure 2, in which *T* stands to the vertex and denotes the collection of n tasks *t*_1_, *t*_2_, …, *t_n_*, and *E* is the collection of directed edges, which denotes the dependency or priority restrictions among jobs in the workflow. A complete graph G = (P, EG) can also be used to depict the cloud-fog network’s processors.

Assume *P_cn_* and *P_fn_* stand for the numerous cloud nodes and fog nodes, respectively. Then, *P = P_cn_∪P_fn_* can be used to represent the total number of nodes. When *pr =* 1, 2, …, *m*, the processing rate and bandwidth of a processor are denoted as *P_prt_[pr*] and *P_ba_[pr]*, respectively. The processing rates of each heterogeneous cloud and fog processor vary. Although the fog nodes are placed in one layer and far from the cloud, their bandwidth is equivalent to that of the cloud nodes because of this. It is assumed that a task that is transferred to the cloud will begin running right away without having to wait in line.

In Figure 2, we take the fog computing processors as *Pf*_1_, *Pf*_2_, …, *Pf_n_* and cloud computing processors as *Pc*_1_, *Pc*_2_, …, *Pc_n_*, with eight dependent tasks (*T*1, *T*2, …, *Tn*) represented in a DAG. The input tasks are initially processed by fog nodes and then by cloud nodes as well as the results of earlier actions. The main goal is to allocate jobs to the processing units in a way that maximizes system performance while minimizing energy and cost usage. Here, our task scheduler takes into account all of these factors when scheduling tasks on processors.

### 4.1. Objective Functions

The main goal of this article is to reduce costs, energy use, and makespan to boost customer happiness and enhance the service provider’s profit. So, the following is how the objective functions are calculated.

#### 4.1.1. Makespan

The length of time it takes to execute a workflow from beginning to end is known as its makespan. As a result, makespan, MKS, is determined as follows:

(1) $$\mathrm{MKS}\;=\max\{FST_{ti},ti\in T\}-\min\{SRT_{ti},ti\in T\}$$

Here *SRT_ti_* and *FST_ti_* stand for task *ti* beginning and concluding times in a workflow, respectively.

#### 4.1.2. Energy Consumption

The active and idle parts of the energy consumption model are represented by *E_act_* and *E_ide_*, respectively. The term “*E_act_*” describes the power used when a job is being performed and “*E_ide_*” refers to the energy spent when a resource is idle. The active energy is determined using

(2) $$E_{act}=\sum_{i=1}^{n}\alpha fr_{i}vl_{i}^{2}(FST_{ti}-SRT_{ti})$$

Here, the supply voltage and frequency for the resources of task *i* is represented by $vl_{i}^{2}$ and *fr_i_*, and α is a constant. The resource enters a state of sleep during the time it is not being used, where the relative frequency and voltage supply level are at their lowest levels. As a result, the following equation is used to calculate the amount of energy used while inactive:

(3) $$E_{ide}=\sum_{j=1}^{m}\sum_{ide_{j}\in IDE_{j}}^{}\alpha fr_{\min j}vl_{\min j}^{2}LN_{j}$$

Here, *fr_minj_* and $vl_{\min j}^{2}$ are denoted as the frequency and lowest supply voltage on resource *j*, correspondingly, and the length of idling time for *ide_j_* is denoted as *LN_j_.* The total amount of energy (*TE*) used by the cloud-fog system to complete the full operation is

(4) $$TE=E_{act}+E_{ide}$$

#### 4.1.3. Computation Cost

There is a monetary cost associated with each work that one computing node completes. There are two components to the computing cost of a given task: processing and memory costs, which can be estimated as follows.

(5) $$CS_{i}^{cmp}=\sum_{j=1}^{m}(cs_{j}^{p}\times E_{ij}+cs_{j}^{m}\times T_{i}^{mem})\times x_{ij},\;\;\forall i\in \{1,\ldots \ldots n\}$$

Here, *x_ij_* is zero or one; if cloud and fog nodes are available for task *ti*, the *x_ij_* value is 1, otherwise the *x_ij_* value is *0*, and $cs_{j}^{m}$ $cs_{j}^{p}$ are constants that indicate the cost of using the RAM and CPU for node *N_j_*, respectively. The amount of main memory needed for task *T_i_* is denoted by $T_{i}^{mem}$. This equation leads to the following definition of the total computing cost *TCmp* for a set of n tasks,

(6) $$TCmp=\sum_{i=1}^{n}CS_{i}^{cmp}$$

#### 4.1.4. Communication Cost

The cost of communication is included for a particular task in addition to the computation cost. The total size of the task’s output and input items as well as the price of bandwidth utilization per node data unit determine this cost. Let $cs_{j}^{b}$ be the node *N_j’_s* cost of bandwidth usage per data unit and $T_{i}^{bdw}$ the task *T_i’_s* bandwidth requirement in bytes. The following is how the task *T_i_* communication cost is determined.

(7) $$CS_{i}^{comu}=\sum_{j=1}^{m}(cs_{j}^{b}\times T_{i}^{bdw})\times x_{ij},\;\forall i\in \{1,\ldots \ldots n\}$$

Consequently, the following equation gives the total cost of communication for all n tasks.

(8) $$TComu=\sum_{i=1}^{n}CS_{i}^{comu}$$

#### 4.1.5. Total Cost

Now we can obtain the total cost using the following equation.

(9) TCost=TComu+TCmp

Finally, from the above description, the objective function is defined as

(10) Obj=min(MKS+TE+TCost)

## 5. Proposed Task Scheduling Algorithm

Due to the numerous parameters and requirements in the objective function, scheduling the task problem in cloud-fog computing is challenging to solve in polynomial time. We combined the benefits of the electric fish optimization (EFO) and earthworm optimization algorithms (EOA) to build a hybrid heuristic algorithm that minimizes the latency of all jobs and lowers the energy consumption of nodes. It fixes the cloud-fog computing environment’s optimal job scheduling complexities.

The fitness value and random sample serve as the basis for the execution of the EEOA algorithm. The objective function of the strategy is connected with a random value that was originally assigned. If *FN_i_* > *rnd*, the EFO algorithm’s active electrolocation is used for position updating; otherwise, EOA is used in place of passive electrolocation to enhance the effectiveness of the suggested strategy.

The position of the electric fish’s prey and its communication patterns are taken into account when developing the EFO algorithm. Here, the search space (cloud-fog environment) in which the electric fish population *N* (number of VMs) is produced is generated randomly (11).

(11) $$fn_{i,j}=fn_{\min j}+\delta (fn_{\max j}-fn_{\min j})$$

In the population of size |*N*|, where *i =* 1, 2, *… |N|* and an arbitrary value is called that ranges among [0, 1], the position of the *i*^th^ element is expressed as *fn_i_*_,*j*_ in the search area. In Equation (11), *fn_minj_* and *fn_maxj_* denote the lower and upper boundaries correspondingly. Based on the fitness value given in Equation (12), an individual’s frequency value (makespan, energy, and cost) is calculated

(12) $$fr_{i}^{t}=fr_{\min}+\left(\frac{FN_{wrst}^{t}-FN_{i}^{t}}{FN_{wrst}^{t}-FN_{bst}^{t}}\right)+(fr_{\max}-fr_{\min})$$

Electric fish are significantly nearer to the food supply, in the range of *fn_minj_* to *fn_maxj_*, and have their frequency values determined at time *t*. The best and worst fitness values, which are determined by individuals in the population at iteration *t*, are denoted as $FN_{bst}^{t}$ and $FN_{wrst}^{t}$, respectively. A probability computation is then performed using the frequency values *fr_max_* and *fr_min_*, which are fixed *t* 0 and 1, respectively. The amplitude of the *i*th element is determined at time *t* utilizing the weight of the individual’s prior amplitudes as indicated in Equation (13).

(13) $$Amp_{i}^{t}=\alpha •Amp_{i}^{t-1}+(1-\alpha )fr_{i}^{t}$$

Here, a constant value is denoted as α, which is located between [0, 1]. Here each individual’s starting frequency value *fr_i_* is allocated to their starting amplitude value. By taking into account each person’s frequency value across all iterations, the population is divided into two sets. The algorithm adheres to “both passive and active electrolocation” energetic electrolocation NActive: The EFO algorithm’s ability to leverage local search is dependent on active electrolocation. In the following equation, the amplitude parameter *Amp_i_* is utilized to compute the active range of each fish.

(14) $$ac_{i}=Amp_{i}(fn_{\max j}-fn_{\min j})$$

To locate nearby individuals (B|B ⊂ N) within the sensing or active range, it is necessary to determine the distance between the *i*^th^ individual and the remainder of the population *N*. The distance (ds) between people *i* and *k* are calculated using the Cartesian distance formula, as stated in Equation (15).

(15) $$ds_{ik}=\left\|fn_{i}-fn_{k}\right\|=\sqrt{\sum_{j=1}^{d}{\left(fn_{ij}-fn_{kj}\right)}^{2}}$$

Equation (16) is utilized if there is just one neighbor in the active sensing field; otherwise, Equation (17) is used.

(16) $$fn_{ij}^{cnde}=fn_{ij}+\delta (fn_{kj}-fn_{ij})$$

(17) $$fn_{ij}^{cnde}=fn_{ij}+\delta •ac_{i}$$

A randomly selected individual, k, is obtained from the *i*th individual neighbor set in Equation (17), where δ∈ [−1, 1], *ds_ik_* ≤ *ac_i_*, and k ∈ B. This $f_{ij}^{cnde}$ is then depicted as a feasible solution for the *i*th individual. The mathematical modeling presented in Equation (18) is utilized, where the *j*th variable value is greater than the problem space border.

(18) $$fn_{ij}^{candidate}=\{\begin{array}{l} fn_{\min j}\;\;\;\;fn_{ij}^{cnde}<fn_{\min j}\\ fn_{ij}^{cnde}\;\;fn_{\max j}>fn_{ij}^{cnde}>fn_{\min j}\\ fn_{\max j}\;\;\;\;fn_{ij}^{cnde}>fn_{\max j} \end{array}$$

The passive electrolocation is updated using the EOA algorithm to boost the performance of the suggested method. It was created using the earthworms’ contribution to nature as its model. Since earthworms are hermaphrodites, a young earthworm can be born to just one parent. Equation (19) has a mathematical formulation of the reproduction process.

(19) $$ER_{l1.m}=ER_{\max.m}+ER_{\min.m}-\eta ER_{l.m}$$

where the earthworm is located, the lower and upper positions of the earthworm are designated by *ER*_max.*m*_ and *ER*_min.*m*_, respectively. The new location of the *l*th earthworm is represented by *ER_l_*_1*.m*_. The number l is denoted by *ER_l.m_*. The symbol stands for the similarity factor, which establishes how far off the parent and child are from one another. When there is little similarity between them, their distance from one another is minimal. When *ER_l_*_1_ is close to *ER_l_*, a local search is conducted. If *η* = 0, there is a significant distance between them, as shown in Equation (20).

(20) $$ER_{l1.m}=ER_{\max.m}+ER_{\min.m}$$

When the similarity factor has a value of *η* = 1(Equation (21)), a global search is initiated. This is also referred to as the optimal-based learning approach.

(21) $$ER_{l1.m}=ER_{\max.m}+ER_{\min.m}-ER_{l.m}$$

In the reproduction process, making adjustments to the value of *η* balances the exploration and exploitation stages. The passive electrolocation is then updated using the EOA method. The tasks are assigned based on the updated value, which also identifies the optimal fitness values.

The EEOA’s pseudo-code is provided by Algorithm 1.
**Algorithm 1**: The EEOA’s pseudo-code**Input:***T_n_*, N, P_cn,_ P_fn_**Output:** optimal mapping of tasks by minimizing *MKS*, *TE*, *TCost*Initialize the population N (no. of VMs)Calculate each individual’s fitness FNDetermine frequency values x (energy, cost, makespan) and amplitude Amp by Equations (12) and (11) for every iteration t ∈ N  if FN_i_ > rnd   updating the solution using the EFO algorithm's active electrolocation   select at random j for the adjustment   Calculate active range ac_i_ by Equation (13).   Compute distance ds_ik_ by Equation (14)   Recruit nearby residents in the B sensing area.    if B ≠ φ    Select a random individual k in the search space    change *j*^th^ parameter using Equation (15)   else    change *j*^th^ parameter using Equation (16)   end if  else   update the passive electrolocation solution based on EOA using Equation (19)  end if   Identify and upgrade the optimum result  end

## 6. Results and Discussion

This section describes the experimental setting and compares our suggested EMCS algorithm to a few other current methods to evaluate it. The algorithms used in this work were tested and put into place using the CloudSim 3.0.3 simulator with Java on a personal computer with an i7-8550U CPU running at 1.80–2.0 GHz (8 Cores), 16 GB of RAM, and the Windows 10 operating system.

The data workflows for the performance assessment of the suggested method are extracted from two real-world supercomputing sites. They are the High-Performance Computing Center North (HPC2N) and Curie supercomputers executed at the CEA research center (CEA-Curie) in Sweden (https://www.cs.huji.ac.il/labs/parallel/workload, accessed on 14 October 2022). The implementation traces generated from the processing of concurrent HPC tasks are contained in such workload logs. Table 2 provides an overview of these workload logs. To shorten the execution duration of the simulations in this investigation, we built 10 workloads using these HPC2N and CEA-Curie task logs.

We take into account both fog and cloud nodes with various processing, cost, and energy rates. The processing speed of each node is determined by its MIPS (million instructions per second) and communication costs. Cloud networks have VMs and servers with increased processing speeds and bandwidth, but the fog nodes have low bandwidth and processing speeds, which places a price on the use of the processors. The fog nodes have a wider bandwidth than the cloud nodes. Grid Dollars (G$) are used to indicate the cost. Table 3 displays the configuration information for the presented work.

### 6.1. Simulation Results

The effectiveness of the implemented task scheduling techniques was compared to other existing scheduling techniques using simulation experiments. These scheduling policies included cuckoo search particle swarm optimization (CSPSO), cuckoo search and differential evolution algorithm (CSDEO), hybrid oppositional differential evolution-enabled whale optimization algorithm (h-DEWOA) [32], and blacklist matrix-based multi-objective algorithm (BLEMO). To eliminate uncertainty from the investigational data acquired, every simulation trial was performed 30 times while maintaining the identical workload and test settings. The average of the 30 measurements was then recorded.

### 6.2. CEA-Curie Workload Results

**Figure 3:**
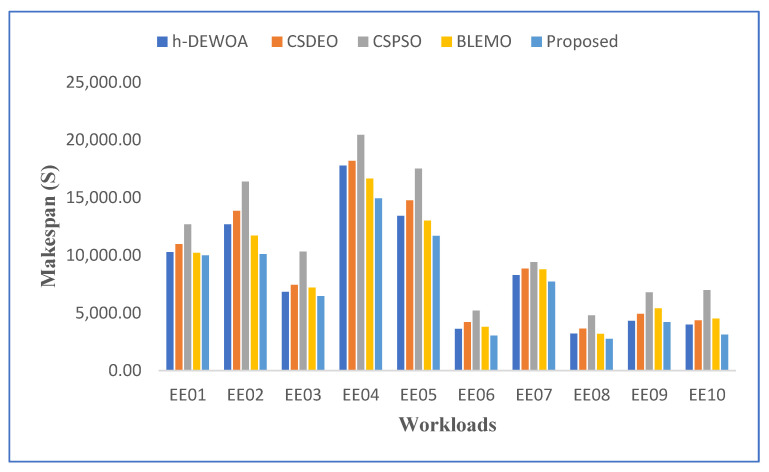
Graphical representation of best makespan values on CEA-Curie workloads.

Table 4 and Figure 3 display the makespan outcome for all scheduling techniques for CEA-Curie workloads. Due to its failure to take advantage of the diversity of virtual machines, CSPSO produced the worst outcomes. CSDEO was also unable to get good results because it did not have a reason for how jobs were assigned and how task orders were made. 

Additionally, it can be shown in Figure 3 that the results obtained by h-DEWOA and BLEMO are often fairly similar and do not exceed the proposed approach. This indicates that the scheduling decisions made by these algorithms regarding the distribution of resources or the sequencing of jobs have little impact on the makespan results. The combination of EFO and EOA, which directs the algorithm toward position updating without compromising the computational cost, is responsible for the performance observed with our suggested approach. This strategy aids in the quickest possible return of the local best answer by our suggested method. Additionally, it contributes to enhancing the quality of solutions at the end of the search process, making it more effective for cloud task scheduling.

**Figure 4:**
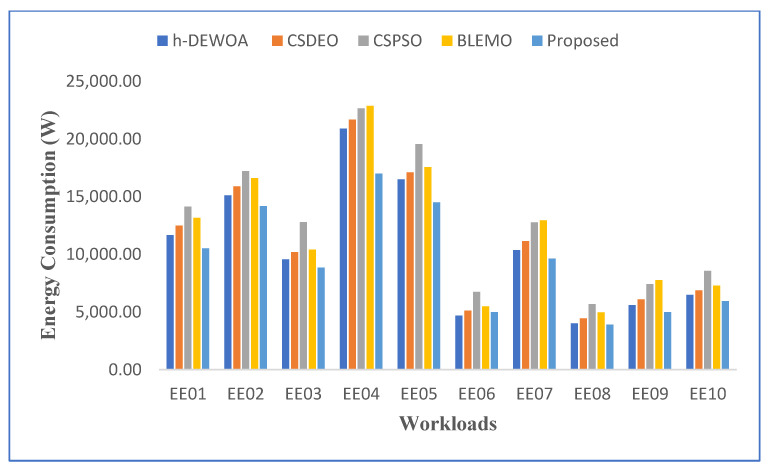
Comparison of energy consumption on CEA-Curie workloads.

A comparison based on energy consumption is shown in Figure 4. The proposed method uses the least amount of energy across all workloads, as would be predicted. H-DEWOA and BLEMO achieve comparable energy usage in all workloads, whereas CSPSO and CSDEO perform poorly in the majority of workloads. The proposed approach achieves significantly more energy consumption on the EE02 and EE05 workloads as contrasted to all other workloads. However, it uses the least amount of energy when compared to existing methods. The CSPSO performs poorly on the EE04 and EE05 tasks, while the CSDEO and H-DEWOA perform similarly. The CSPSO records the highest overall energy use. This further demonstrates how unreliable and ineffective the CSPSO algorithm is. It uses a single homogeneous population, which is the cause of this. Thus, the potential for early convergence is always present.

**Figure 5:**
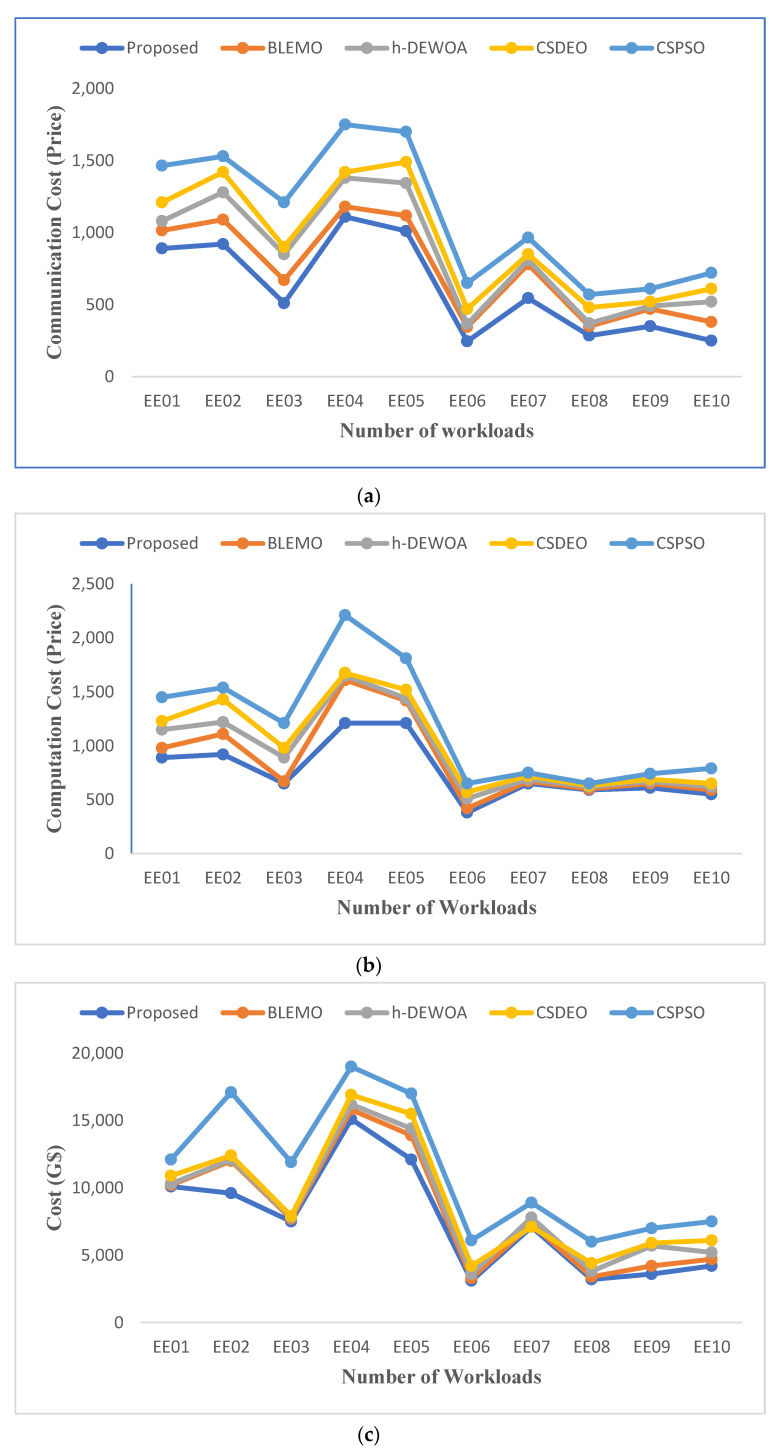
Comparison of (**a**) communication cost, (**b**) computation cost, and (**c**) total cost of CEA-Curie workload.

Figure 5 shows the cost of communication, calculation, and overall execution. These figures show that each job has a different total execution cost. The presented EEOA task scheduling algorithm operates better and outperforms the h-DEWOA, CSDEO, CSPSO, and BLEMO task allocation algorithms in terms of minimizing execution costs. The results obtained further demonstrate the scalability and ability of the suggested approach to schedule large jobs in a heterogeneous environment while incurring the minimum execution costs. For EE01 and EE10 workloads, our proposed EEOA algorithm improves the quality of its solutions by allocating tasks to the finest VMs with the lowest execution costs.

### 6.3. HPC2N Workload Results

**Figure 6:**
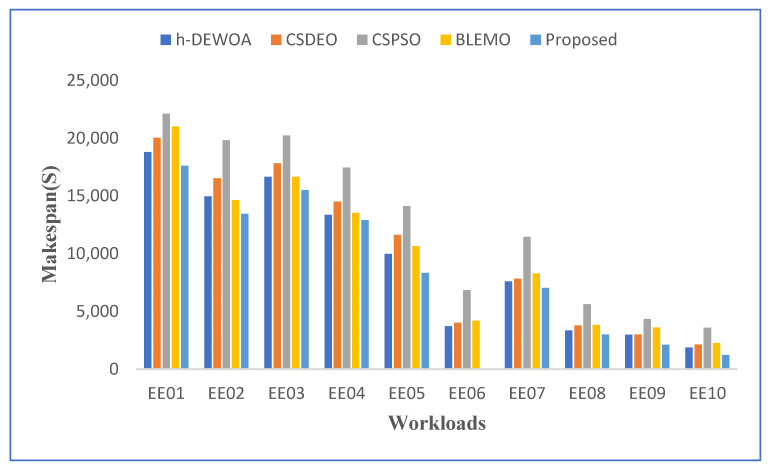
Best makespan result for HPC2N workload.

Results for the HPC2N workload in terms of makespan, cost, and energy usage are shown in this section. Table 5 and Figure 6 show the makespan results of the proposed methodology compared with other algorithms. It shows how much better makespan is than the existing algorithms for workload situations. This is because the EOA algorithm has been hybridized into the EFO algorithm, which provides a balance between global and local search, improving the result globally.

The graphical representation of the best makespan results for all scheduling strategies is shown in Figure 6 and was obtained by running the EE01–EE10 workloads of the HPC2N trace. The graph makes it clear that, out of all scheduling strategies, the proposed technique leads to the lowest makespan findings. It provides compelling evidence of the proposed scheme’s stability and robustness. The worst outcomes, which include unanticipated terrible behavior, are produced by other meta-heuristic techniques such as CSPSO and BLEMO, which are notably different from the others. However, compared to these methods, the h-DEWOA meta-heuristic produced better results.

**Figure 7:**
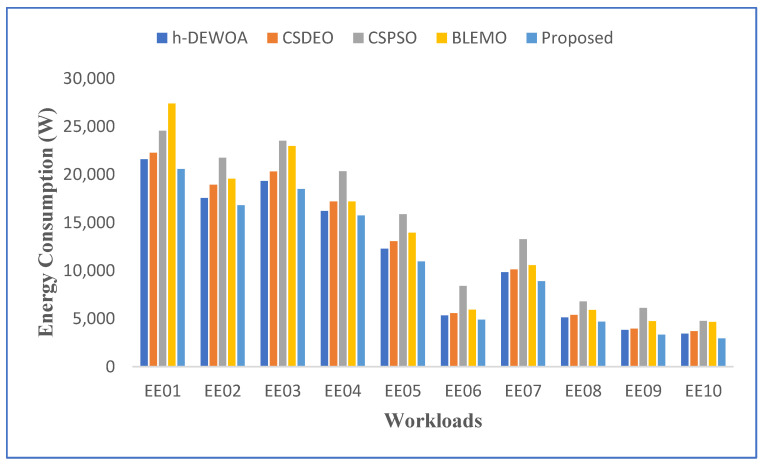
Comparison of energy consumption for HPC2N workload.

The same evaluation was carried out to see how much energy the strategies saved. When employing EEOA, the system used 10% less energy than when using the other approaches for the HPC2N workloads in Figure 7. This implies that it reduced both energy and food consumption. This is because of an efficient hybrid mechanism that combines the EFO and EOA algorithms’ superior searching capabilities. The suggested hybrid policy had access to effective and varied schedules at every generation owing to hybridization, and the EEOA algorithm’s final iterations showed no loss of variety.

**Figure 8:**
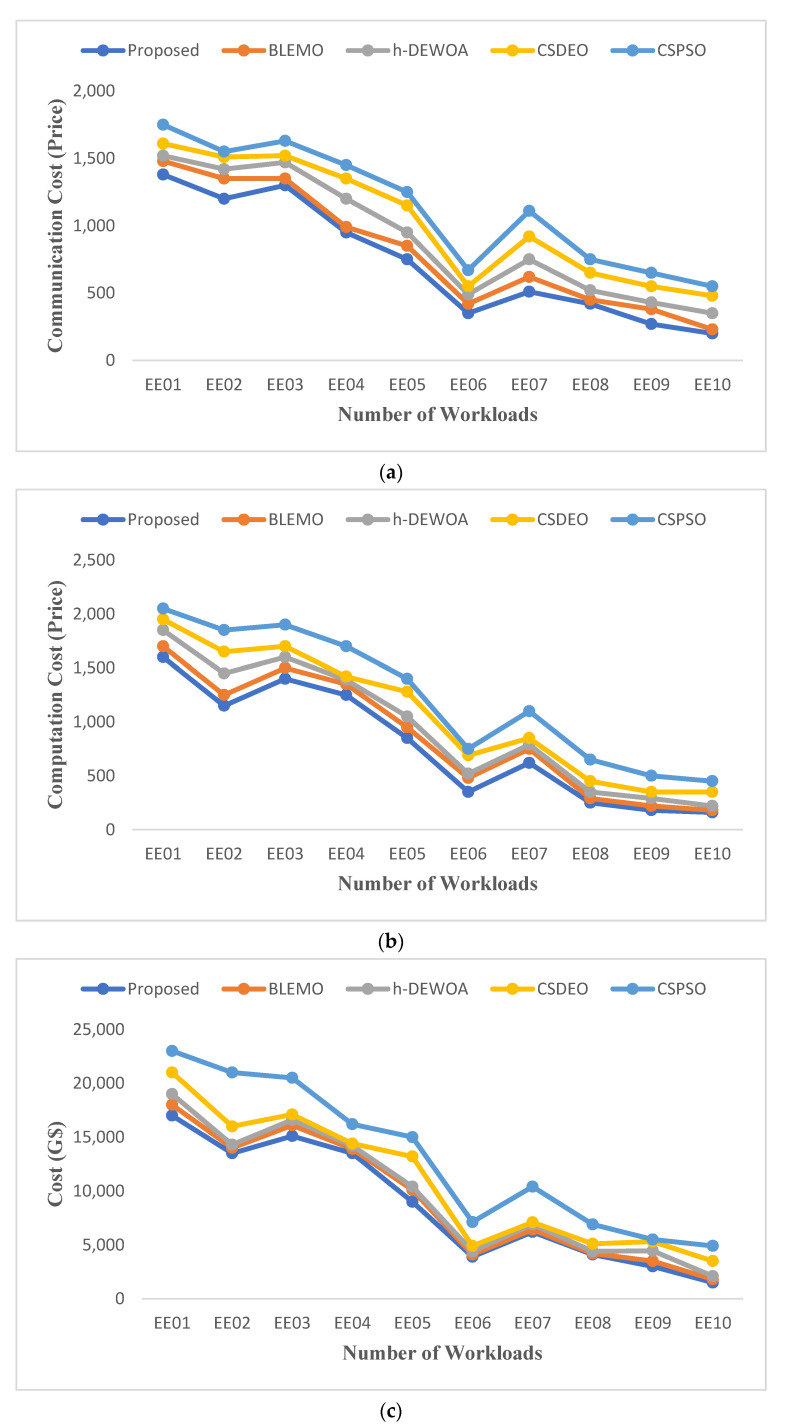
Comparison of (**a**) communication cost, (**b**) computation cost, and (**c**) total cost of HPC2N workload.

The entire amount that the clients pay to the service supplier depending on the number of resources utilized is known as the execution cost. Figure 8 displays the communication cost, computation cost, and total cost figures for the various algorithms. The EEOA algorithm can reduce the cost for a variety of workloads while improving performance more than any other method.

It has been noted that CSPSO has the highest cost, EEOA has the lowest cost, and CSDEO and BLEMO have the average cost of using cloud and fog for EE01 and EE10 workloads, as illustrated in Figure 8. While ignoring additional prices and energy, CSPSO concentrates on the task’s processing time. Therefore, its price is the highest. h-DEWOA can save more on average cost when compared to CSDEO; it can save 25.38% of the average cost of CSPSO and 3.5% of the average cost of CSDEO. This is because the suggested technique shortens overall execution time by assigning the work to the resource that can perform it most quickly, which can also help lower costs.

As demonstrated in Table 6, the suggested EEOA approach generated good scheduling solutions with appropriate convergence speeds for both workloads. The suggested scheduling policy’s time requirements are lower than those of the h-DEWOA and CSDEO meta-heuristic-based scheduling strategies. The computation time of the h-DEWOA algorithm is quite higher than that of other techniques. However, it is feasible despite the significant makespan, cost, and energy consumption. Finally, the proposed methodology gives the best results in terms of efficiency, cost, and energy usage with the least amount of computing time when compared to the other methods. As a result, it can be said that the EEOA has proven to be a potent and successful solution for resolving complex task-scheduling issues on a large scale.

## 7. Conclusions and Future Work

The management of the allocation and execution of IoT jobs in a cloud-fog computing environment proves the effectiveness of the cost-aware task scheduling system. To solve the job scheduling problem, we suggest a novel adaptive algorithm in this article that integrates the earthworm optimization algorithm (EOA) with the electric fish optimization algorithm (EFO). The goal of the provided EEOA approach was to increase the fundamental EFO algorithm’s capacity for use. The effectiveness of the proposed algorithm was assessed using several assessment criteria and compared to that of the other metaheuristics already in use. The presented scheduling method performs better than alternative methods in general across all scientific workflows and all performance measures according to the results. It holds its own against other procedures reasonably well and produces the best outcomes in the shortest amount of time. The proposed mechanism is applied in real-time applications, i.e., smart cities, and it also helps greatly in scheduling latency sensitive applications such as vehicular networks. One of the limitations we discovered in our current research is that our algorithm cannot predict upcoming workloads and cannot decide whether or not to offload the task. As a result, in the future, the scheduling algorithm will need to incorporate a machine learning model that predicts upcoming workloads and intelligently offloads tasks at the fog nodes. Furthermore, we will test our proposed work in an edge-cloud environment to determine the efficiency of our algorithm.

## Figures and Tables

**Table 1:** Analysis of scheduling parameters in the related works.

Authors	Technique Used	Parameters Addressed
[26]	GA-IRACE	Execution time
[27]	GWOTS	Makespan, cost, energy consumption
[28]	RACE	Execution time, bandwidth
[29]	ECBTSA-IRA	Schedule length, cost, energy
[30]	MS-PSO	Load balance, power consumption, computation time
[31]	MQP	Latency
[32]	h-DEWOA	Task duration, energy consumption
[38]	MOTSWAO	Makespan, SLA-based trust parameters, energy consumption
[39]	TAFFA	Makespan, SLA-based trust parameters.
Proposed algorithm	EEOA	Makespan, total cost, and energy consumption

**Table 2:** Description of real workloads.

Workload Log	Duration	Parallel Tasks	Users	CPUs	File
HPC2N	July 2002–January 2006	202,871	257	240	HPC2N-2002-2.2-cln.swf
CEA-Curie	February 2011–October 2012	312,826	582	93,312	CEA-Curie-2011-2.1-cln.swf

**Table 3:** Configuration details.

Parameter	Cloud	Fog	Unit
Number of VMs	[10, 15, 20]	[15, 20, 35]	VM
Computing power	[3000:5000]	[1000:2000]	MIPS
RAM	[5000:20000]	[250:5000]	MB
Bandwidth	[512:4096]	[128:1024]	Mbps
Cost	[0.6:1.0]	[0.2:0.5]	G$

**Table 4:** Makespan results for CEA-curie workloads.

CEA-Curie Workloads	Statistics	h-DEWOA	CSDEO	CSPSO	BLEMO	Proposed
EE01	Best	10,275.90	10,969.92	12,675.80	10,200.10	9987.45
	Average	11,660.04	12,482.34	14,134.60	13,165.61	10,526.87
	Worst	14,435.67	15,499.38	16,988.50	20,732.08	12,498.12
EE02	Best	12,679.97	13,850.81	16,376.80	11,700.21	10,102.87
	Average	15,109.39	15,889.18	17,221.90	16,597.74	14,187.21
	Worst	18,477.35	18,831.50	20,454.50	24,840.16	16,932.67
EE03	Best	6829.378	7442.08	10,311.67	7200.21	6456.78
	Average	9560.564	10,186.98	12,798.60	10,413.30	8843.67
	Worst	11,850.62	13,173.06	15,224.90	16,350.10	10,631.33
EE04	Best	17,764.81	18,177.46	20,445.71	16,650.21	14,933.78
	Average	20,896.71	21,676.68	22,658.40	22,875.85	17,005.87
	Worst	24,003.90	25,609.87	26,113.80	32,400.10	20,947.23
EE05	Best	13,416.31	14,758.71	17,523.90	13,005.17	11,673.20
	Average	16,508.44	17,108.33	19,543.20	17,552.51	14,498.56
	Worst	20,937.46	19,959.83	22,665.43	24,444.68	17,837.98
EE06	Best	3620.954	4203.09	5209.51	3798.96	3046.78
	Average	4684.121	5109.55	6754.31	5483.11	4991.76
	Worst	6140.261	6733.47	8991.11	8535.21	5821.65
EE07	Best	8277.555	8843.01	9411.80	8771.46	7712.96
	Average	10,371.22	11,153.84	12,774.12	12,950.13	9623.12
	Worst	12,517.62	13,390.13	15,934.89	20,400.21	11,178.39
EE08	Best	3208.096	3653.73	4788.31	3180.21	2749.81
	Average	4008.443	4451.31	5683.80	4967.06	3912.78
	Worst	6041.973	5876.96	6371.51	8325.21	5129.21
EE09	Best	4308.133	4929.97	6783.11	5400.1	4200.12
	Average	5599.78	6093.44	7416.8	7770.17	4984.98
	Worst	7020.00	8509.48	9003.7	14,850.21	6793.76
EE10	Best	3988.775	4368.56	6987.6	4500.21	3122.56
	Average	6481.927	6876.61	8564.9	7282.67	5952.34
	Worst	7672.408	7924.87	10,277.6	11,250.10	6932.77

**Table 5:** Makespan results for HPC2N workload.

HPC2N Workloads	Statistics	h-DEWOA	CSDEO	CSPSO	BLEMO	Proposed
EE01	Best	18,801.74	20,036.63	22,109.82	21,000.10	17,612.91
	Average	21,579.70	22,258.71	24,562.89	27,397.33	20,561.23
	Worst	33,700.98	35,291.42	27,988.71	41,259.62	26,333.98
EE02	Best	14,953.21	16,522.34	19,822.77	14,644.01	13,451.72
	Average	17,567.38	18,941.76	21,753.88	19,552.95	16,812.56
	Worst	22,324.56	22,696.08	24,123.77	28,848.91	20,712.40
EE03	Best	16,649.19	17,810.82	20,237.12	16,650.10	15,512.89
	Average	19,329.17	20,308.97	23,521.98	22,961.31	18,490.21
	Worst	26,644.20	29,312.09	25,988.25	33,907.60	22,651.48
EE04	Best	13,366.98	14,515.07	17,452.86	13,522.67	12,911.47
	Average	16,207.58	17,188.48	20,343.71	17,200.76	15,741.49
	Worst	20,203.84	20,332.90	22,329.21	22,965.20	18,490.28
EE05	Best	9972.00	11,616.94	14,117.48	10,650.25	8328.61
	Average	12,274.21	13,067.45	15,876.22	13,953.94	10,953.47
	Worst	14,380.36	14,915.63	16,432.21	18,150.31	13,723.91
EE06	Best	3704.70	4017.70	6843.91	4200.10	3208,11
	Average	5320.46	5576.38	8410.39	5930.53	4879.94
	Worst	7980.15	7468.60	9988.78	11,625.10	6247.56
EE07	Best	7581.52	7828.44	11,447.30	8287.76	7032.81
	Average	9821.84	10,110.14	13,256.89	10,553.76	8892.02
	Worst	12,118.86	11,991.02	14,952.12	15,232.71	10,871.45
EE08	Best	3338.51	3778.44	5623.54	3825.10	2988.52
	Average	5137.35	5380.60	6782.13	5910.08	4693.29
	Worst	7803.77	7980.22	8705.65	9450.17	7302,27
EE09	Best	2969.94	3006.86	4336.83	3600.10	2100.82
	Average	3834.73	3964.54	6123.98	4738.25	3319.82
	Worst	5808.41	7878.97	7398.21	21,937.59	4847.62
EE10	Best	1875.20	2130.94	3581.54	2250.10	1211.39
	Average	3436.67	3697.48	4769.21	4657.41	2931.73
	Worst	5548.10	7232.97	7932.42	20,970.09	4831.29

**Table 6:** Execution time for two workloads.

Techniques	CEA-CURIE (s)	HPC2N (s)
h-DEWOA	14.87	15.59
CSDEO	11.63	15.97
CSPSO	10.73	15.07
BLEMO	7.95	11.47
Proposed	7.12	10.86

## Data Availability

Data will be made available on request.
